# U-Shape Relationship between Plasma Leucine Level and Mortality in the Intensive Care Unit

**DOI:** 10.1155/2022/7389258

**Published:** 2022-01-07

**Authors:** Mei-Ying Wang, Chao-Hung Wang, Wei-Siang Chen, Chien-Ming Chu, Huang-Ping Wu, Min-Hui Liu, Yi-Tsen Lin, Kuo-Chin Kao, Chung-Yu Liang, Wen-Hsin Chen, Huei-Jen Wang, Shu-Chiu Lee

**Affiliations:** ^1^Heart Failure Research Center, Division of Cardiology, Department of Internal Medicine, Chang Gung Memorial Hospital, 20401 Keelung, Taiwan; ^2^Intensive Care Unit, Division of Cardiology, Department of Internal Medicine, Chang Gung Memorial Hospital, 20401 Keelung, Taiwan; ^3^Chang Gung University College of Medicine, 33302 Taoyuan, Taiwan; ^4^Division of Pulmonary, Critical Care and Sleep Medicine, Chang Gung Memorial Hospital, 20401 Keelung, Taiwan; ^5^Department of Nursing, Keelung Chang Gung Memorial Hospital, 20401 Keelung, Taiwan; ^6^Nutrition Department, Chang Gung Memorial Hospital, 20401 Keelung, Taiwan; ^7^Department of Thoracic Medicine, Chang Gung Memorial Hospital, 33305 Taoyuan, Taiwan

## Abstract

Patients in the intensive care unit (ICU) are at high risk of mortality which is not well predicted. Previous studies noted that leucine has prognostic value in a variety of diseases. This study investigated whether leucine concentration was a useful biomarker of metabolic and nutritional status and 6-month mortality in ICU. We recruited 454 subjects admitted to ICU (348 and 106 in the initiation and validation cohorts, respectively) with an acute physiology and chronic health evaluation (APACHE II) score ≥ 15. We measured plasma leucine concentrations, traditional biomarkers, and calculated APACHE II and sequential organ failure assessment (SOFA) scores. Leucine levels were weakly correlated with albumin, prealbumin, and transferrin levels (*r* = 0.30, 0.12, and 0.15, *p* = 0.001, 0.029, and 0.007, respectively). During follow-up, 116 (33.3%) patients died. Compared to patients with leucine levels between 109 and 174 *μ*M, patients with leucine > 174 *μ*M or <109 *μ*M had a lower cumulative survival rate. Death was also associated with age, higher APACHE II and SOFA scores, C-reactive protein, and longer stays in the ICU, but with lower albumin, prealbumin, and transferrin. Patients with leucine levels > 174 *μ*M had higher alanine aminotransferase levels, but no significant differences in other variables; patients with leucine levels < 109 *μ*M had higher APACHE II and SOFA scores, higher incidence of using inotropic agents, longer ICU and hospital stays, but lower albumin and transferrin levels. Multivariable analysis demonstrated that leucine > 174 *μ*M was an independent predictor of mortality, especially early mortality. However, among patients who stayed in ICU longer than two weeks, leucine < 109 *μ*M was an independent predictor of mortality. In addition, leucine < 109 *μ*M was associated with worse ventilator weaning profiles. These findings were similar in the validation cohort. Our study demonstrated a U-shape relationship between leucine levels and mortality rate in ICU.

## 1. Introduction

Patients admitted to the intensive care unit (ICU) are at high risk of morbidity and mortality [1–3]. Catabolic status and/or malnutrition are associated with poor outcomes. In 1950, Cuthbertson et al. defined distinct catabolic pathophysiology and nutritional deficiency at different phases throughout the trajectory of critical illness [4]. To mitigate these devastating metabolic disturbances, assessment of patients' catabolic and nutritional status is required for interventions such as protein supplementation or anabolism-stimulating medications. A simple and timely surrogate biomarker covering both catabolic and nutritional status and prognostic value is thus a critical unmet need, especially for patients in a contemporary ICU with overlapping and cyclical phases of metabolism that were not adequately addressed by Cuthbertson et al. long ago, whose work was based on bed-ridden patients with femoral fractures [5].

Nutritional parameters, including albumin, prealbumin, and transferrin, provide prognostic value in critical patients [6–9]. However, all these nutritional biomarkers are proteins with a complex process of synthesis that depends on the amount of amino acids present; moreover, a variety of factors may interfere with the level of these biomarkers, such as inflammation, caloric intake, and iron storage. Leucine, an essential amino acid, is an element of albumin and prealbumin [10, 11]. Previously, we demonstrated that blood leucine concentrations had a clear correlation with the total amount of essential amino acids, indicating that leucine can potentially represent the quantity of fundamental elements for protein synthesis [12]. Moreover, leucine has been proven to have prognostic value in patients with advanced cardiovascular diseases [12]. Thus, further investigation is merited to understand the value of measuring blood leucine levels in patients with critical illness.

As shown in our previous study of patients with heart failure, the J-shape relationship between leucine level and disease outcomes suggests that leucine might indicate catabolic and malnutrition status associated with outcomes [12–15]. However, how to use leucine levels in a clinical setting remains to be elucidated, especially in critical illness. In this study, we hypothesized that plasma leucine concentration could be used as a biomarker of metabolic and nutritional status and mortality in patients cared in the ICU. The aims of this study were (1) to explore the relationship between blood concentrations of leucine and traditional nutrition parameters, (2) to estimate whether leucine provides prognostic value compared to the other traditional parameters, and (3) to understand how leucine level predicts mortality in patients admitted to the ICU for future clinical application.

## 2. Material and Methods

### 2.1. Patient Enrollment

From April 2017 to February 2020, for the initiation cohort, patients were consecutively enrolled at the contemporary medical and cardiac ICU based on these inclusion criteria: (1) they were at critical status with an APACHE II score ≥ 15, (2) they needed to stay in the ICU > 48 hours, and (3) they were older than 20 years old. The exclusion criteria included patients with comorbid disorders other than the main cause for admission that might compromise their survival within three months, such as terminal stage cancer. All patients provided informed consent. The study was designed and carried out in accordance with the principles of the Declaration of Helsinki and with approval from the Ethics Review Board of Chang Gung Memorial Hospital.

**Figure 1:**
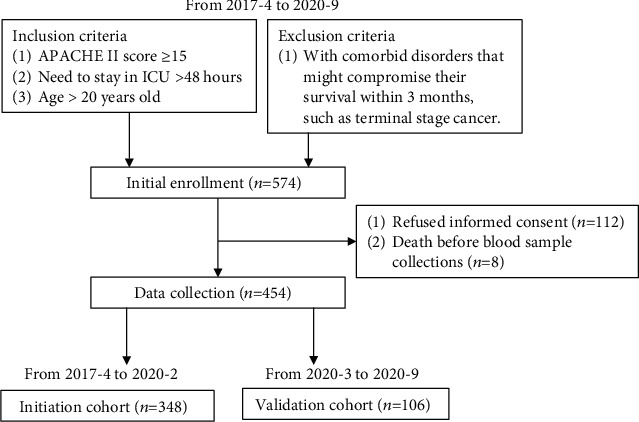
The flow diagram of the study.

The validation phase set, another independent population including 106 patients, was recruited from March 2020 to September 2020 (Figure 1).

### 2.2. Scoring Systems

Disease severity was assessed by SOFA scores [16] on the first day of admission to the intensive care unit. APACHE II scores [2] were calculated both on the first day and 2 weeks later after admission to the ICU.

### 2.3. Blood Sampling and Examination

Fasting blood samples were collected in EDTA-containing tubes in the early morning, the day after obtaining informed consent. Plasma leucine was measured by ultraperformance liquid chromatography (UPLC) workflow. Measurement of other parameters, including prealbumin, transferrin, albumin, estimated glomerular filtration rate (eGFR), C-reactive protein, and hemoglobin, was performed in the central laboratory.

### 2.4. Leucine Measurement

Plasma leucine concentration was quantified by UPLC. Plasma samples (100 *μ*L) were precipitated with sulfosalicylic acid (10%). After protein precipitation and centrifugation, derivatization was conducted by AQC in acetonitrile. Amino acids were analyzed using the ACQUITY UPLC System, consisting of a Sample Manager, a Binary Solvent Manager, and a Tunable UV detector. EmpowerTM 2 Software was used to control the system and collect data. Separations were conducted on a 2.1 × 100 mm ACQUITY BEH C18 column at a flow rate of 0.70 mL/min. The average intra-assay coefficient of variation was 4.5%. The total coefficient of variation was 4.1%, and the detection limit was 0.9 *μ*M. The linear range was 25–500 *μ*M.

### 2.5. Measurement of Respiratory Profile

Respiratory profile was estimated within 2 days after admitted to the ICU if the patients were intubated. The content of the profile included respiratory rate, tidal volume, minute ventilation, and maximal inspiratory pressure which were measured with a Mechanics monitor (Respira-dyne II, Sherwood/Covidien, Boulder, Colorado). The rapid shallow breathing index was calculated as respiratory rate divided by tidal volume and recorded as an index for the pulmonary mechanics and weaning procedure.

### 2.6. Follow-Up Program

Follow-up data were prospectively obtained from hospital records, personal communication with the patients' physicians, telephone interviews with patients, and patients' regular visits to staff physician outpatient clinics. Patients were followed until death or a maximum of 6 months. The primary endpoint was death from all causes. In addition, we analyzed the length of stay in ICU and hospital.

### 2.7. Statistical Analyses

Results are expressed as the mean ± SD for variables with normal distribution, as the median (interquartile range (IQR)) for variables with skewed distribution, and as the number (percentage) for categorical variables. We compared data using *t*-test, one-way ANOVA (with post hoc analysis by Bonferroni), Chi-square (multiple comparison with Bonferroni-adjusted *p* values), the Mann–Whitney *U* test, and Kruskal-Wallis *H* test, when appropriate. We estimated receiver operating characteristic (ROC) curves and used Youden's index to identify the cutoff value of variables. A univariate Cox proportional hazards model was used to determine the variables' predictive value on mortality. To compare time-dependent outcomes, we performed Kaplan-Meier analyses with a log-rank test. Variables with a *p* value < 0.05 in univariate analysis were included in the multivariable analysis. By COX multivariable analysis, we identified independent predictors of mortality by adjusting for significant demographic variables, or by both significant demographic and laboratory variables based on univariate analysis. Hazard ratios (HRs) and 95% confidence intervals (CIs) were calculated. All statistical analyses were two-sided and performed using SPSS software (version 22.0, SPSS, Chicago, IL, USA) and R software (version 3.5.1). A *p* value of < 0.05 was considered significant.

## 3. Results

### 3.1. Baseline Characteristics and Laboratory Data

In the initiation cohort, we enrolled 348 patients. These patients were admitted to the ICU for the following conditions: (1) 157 patients (45.1%) had coronary artery disease, myocardial infarction, or cardiovascular diseases; (2) 92 patients (26.4%) had infection; (3) 44 patients (12.6%) had chronic obstructive pulmonary disease; (4) 15 patients (4.3%) had gastrointestinal bleeding; (5) 7 patients (2.0%) had kidney disease; (6) 5 patients (1.4%) were admitted for sudden collapse; (7) 2 patients (0.6%) for stroke, and 26 patients (7.4%) for other conditions. The average age was 71.4 years, and the average APACHE II and SOFA scores were 20.2 and 7.07, respectively (Table 1). Generally, the enrolled patients had multiple comorbidities, high C-reactive protein levels, but low hemoglobin, cholesterol, triglyceride, albumin, eGFR, prealbumin, and transferrin levels. The validation cohort included 106 patients. The demographic data are shown in the supplemental Table S1.

### 3.2. Relationship between Leucine Levels and Mortality

**Figure 2:**
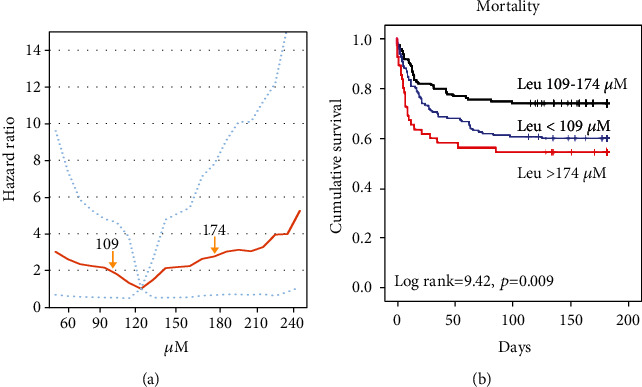
Prognostic value of plasma leucine concentration. (a) Association between leucine level and risk of mortality in the additive Cox regression models (dotted lines indicate 95% confidence interval). (b) The Kaplan-Meier curves for three different groups (for all-cause death).

During the 6-month follow-up period, 116 (33.3%) patients died. In 61 (52.6%) patients, death occurred due to infection, 23 (19.8%) died of critical cardiovascular conditions, 19 (16.4%) due to both conditions combined, 7 (6.0%) due to pulmonary diseases, and 5 (4.3%) due to other reasons. Leucine levels were weakly correlated with albumin, prealbumin, and transferrin levels (*r* = 0.30, 0.12, and 0.15, with *p* = 0.001, 0.029, and 0.007, respectively). Association between leucine level and mortality risk was shown in the additive Cox regression models (Figure 2(a)). Although lower leucine levels were significantly associated with higher mortality rates, higher leucine levels also correlated with higher mortality rates, represented by a U-shaped curve.

Patients were separated into three groups based on the ROC curve analyses and Youden's index consisting of patients with baseline leucine levels < 109 *μ*M, between 109 and 174 *μ*M, and > 174 *μ*M (Supplemental Figure S1). In Figure 2(b), the Kaplan-Meier curves demonstrate that patients with leucine levels > 174 *μ*M or <109 *μ*M had a lower accumulative survival rate, compared to those with leucine levels of 109 to 174 *μ*M (log rank = 9.11 and 6.22, *p* = 0.003 and 0.013, respectively). The mortality rate was 45.5%, 40.0%, and 25.9% in patients with leucine levels > 174 *μ*M and <109 *μ*M and between 109 and 174 *μ*M, respectively. The validation cohort confirmed these findings (Kaplan-Meier curves are shown in supplemental Figure S2).

Patients with leucine levels < 109 *μ*M had higher APACHE II and SOFA scores compared to patients with leucine levels between 109 and 174 *μ*M, higher incidence of using inotropic agents, and longer stays in the ICU and hospital, but lower hemoglobin, cholesterol, triglyceride, albumin, and transferrin levels (Table 2). Patients with leucine levels > 174 *μ*M had higher levels of alanine aminotransferase, but no significant differences in other parameters. In addition, compared to patients with leucine levels < 109 *μ*M, patients with leucine levels > 174 *μ*M had similar prealbumin levels, but higher cholesterol, triglyceride, alanine aminotransferase, albumin, and transferrin levels. The demographic and laboratory data in patients with different leucine concentrations in the validation cohort are shown in supplemental Table S2.

### 3.3. Factors Associated with Mortality

Mortality was associated with age, higher APACHE II and SOFA scores, higher incidence of using inotropic agents, higher C-reactive protein levels, and longer length of stay in the ICU, but also with lower incidence of chronic obstructive pulmonary disease, lower cholesterol, albumin, prealbumin, and transferrin levels (Table 1). In addition, leucine levels between 109 and 174 *μ*M were related to lower mortality rate in both the initiation and validation cohorts (Table 1 and Supplemental Table S1).

### 3.4. High Leucine Predicted Early Mortality

Of the 25 patients who had leucine levels > 174 *μ*M and died in 6 months, 20 (80%) died within the 2 weeks following enrollment. COX univariate analysis showed that mortality within 6 months was associated with leucine > 174 *μ*M or <109 *μ*M, age, APACHE II and SOFA scores, chronic obstructive pulmonary disease, and levels of C-reactive protein, cholesterol, albumin, prealbumin, and transferrin (Table 3). Next, we conducted multivariable analysis by adjusting for significant demographic variables and by adjusting for significant demographic and laboratory variables according to univariate analysis. Either after adjusting for age and chronic obstructive pulmonary disease or after adjusting for age, chronic obstructive pulmonary disease, C-reactive protein, cholesterol, albumin, pre-albumin, and transferrin, leucine > 174 *μ*M remained an independent predictor of mortality within 6 months. Furthermore, even after adjusting for all significant variables in the univariate analysis additionally including APACHE II and SOFA scores, leucine > 174 *μ*M was still demonstrated to be an independent predictor (hazard ratio = 1.97, 95%CI = 1.16 − 3.33, *p* = 0.012).

### 3.5. Low Leucine Predicted Mortality in Patients Who Survived Longer than Two Weeks

Multivariable analysis showed that leucine < 109 *μ*M was an independent predictor of mortality within 6 months after adjusting for significant demographic variables, including age and chronic obstructive pulmonary disease, according to univariate analysis (Table 3). However, when adjusting for significant demographic and laboratory variables, including age, chronic obstructive pulmonary disease, and nutritional biomarkers, the prognostic value of leucine < 109 *μ*M became insignificant.

**Figure 3:**
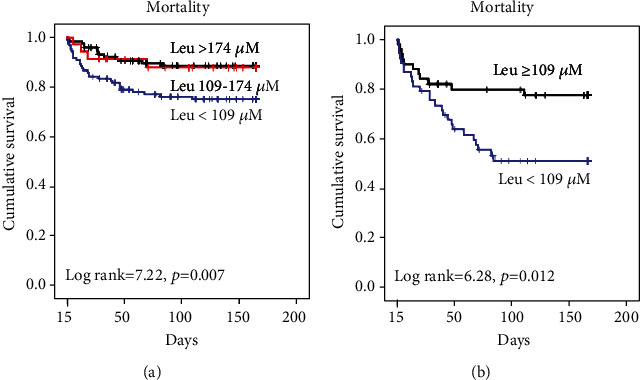
Prognostic value of plasma leucine concentration in patients who survived longer than two weeks. (a) The Kaplan-Meier curves for three different groups (for all-cause death). Leucine levels were measured at baseline. (b) The Kaplan-Meier curves for patients with leucine < 109 *μ*M versus ≥109 *μ*M (for all-cause death). Leucine levels were measured at two weeks after admission to the intensive care unit in patients who remained in the intensive care unit.

In Figure 2(b), comparing patients with leucine levels between 109 and 174*μM* and those with leucine levels < 109 *μ*M, the Kaplan-Meier curves show that the mortality rate did not differ in 2 weeks (log rank = 0.46, *p* = 0.49) but began to diverge after 2 weeks. Thus, we performed the secondary analysis. Of patients who survived longer than 2 weeks, those with leucine levels < 109 *μ*M at baseline had a significantly lower survival rate compared to those with leucine levels between 109 and 174 *μ*M (log rank = 7.22, *p* = 0.007) (Figure 3(b)). Survival rates were similar between patients with leucine between 109 and 174 *μ*M and those with leucine > 174 *μ*M. Compared to patients with baseline leucine levels between 109 and 174 *μ*M, patients with a leucine level < 109 *μ*M at baseline had higher respiratory rate (23.2 ± 7.24 breaths/min vs. 19.9 ± 6.99 breaths/min, *p* = 0.003), rapid shallow breathing index (69.0 (45, 92) vs. 53.5 (33, 75.3), *p* = 0.003), but lower tidal volume (358 ± 158 ml vs. 424 ± 210 ml, *p* = 0.027) in the respiratory weaning profile at baseline and longer length of ICU and hospital stay (Table 2).

Based on these results, we further investigated the prognostic value of leucine concentrations in patients who survived longer than two weeks and were still in the ICU (*n* = 103). Leucine levels were measured again at two weeks after enrollment. In Figure 3(b), Kaplan-Meier curves show that leucine < 109 *μ*M at 2 weeks was significantly associated with a lower survival rate, compared to leucine ≥ 109 *μ*M (log rank = 6.28, *p* = 0.012). COX univariate analysis showed that leucine < 109 *μ*M significantly predicted mortality (hazard ratio = 2.41, 95%CI = 1.18 − 4.89, *p* = 0.016). The prognostic value of leucine < 109 *μ*M was independent of age and APACHE II at two weeks (hazard ratio = 2.34, 95%CI = 1.15 − 4.76, *p* = 0.020).

## 4. Discussion

Our study reveals that the amino acid, leucine, can be used as a biomarker of disease outcomes. Interestingly, we noted a U-shaped relationship between leucine levels and mortality rates; both lower and higher leucine levels predicted higher mortality rates. In addition, low leucine levels became the main prognostic factor when patients stayed in the ICU longer than two weeks. The validation cohort confirmed these findings.

Albumin and prealbumin are two widely used biomarkers of nutrition. Low levels of albumin and prealbumin have been shown to indicate poor outcomes in critical patients [6–8]. However, these biomarkers represent the capacity for protein synthesis associated with nutritional status, and thus, their clinical interpretation is limited by the speed of synthesis and their relatively long half-life (12 and 3 days for albumin and prealbumin, respectively). Leucine is an essential component for albumin and prealbumin synthesis [10, 11], and its blood concentration is assumed to represent the amount of elementary resources for protein synthesis. Intriguingly, our study demonstrated that low albumin and prealbumin levels do not equal low leucine levels. Since both lower and higher leucine levels were associated with lower levels of albumin and prealbumin and a higher mortality rate, albumin and prealbumin-based strategies of nutritional support should be modified further.

The concept of ebb and flow phases is based on Cuthbertson and colleagues' observations on the catabolic state in response to serious injury and other pathophysiological conditions [4, 5]. Most situations we encounter in the contemporary medical ICU involve the acute and adaptive responses during the flow phase. However, these phases and responses often happen repeatedly and may overlap, making them difficult to distinguish by clinical judgement. It is also impractical to identify different metabolic states by performing an instant immune panel or estimating protein synthesis and breakdown using the indicator amino acid oxidation method.

Our data provide a solution for these complex and interdependent metabolic states. Based on existing literature, both protein breakdown and synthesis increase remarkably in response to the tremendous stress at the acute catabolic phase [3, 5]. However, protein synthesis often cannot catch up with the speed of protein breakdown, since a substantial portion of the amino acids released from skeletal muscle, such as branched chain amino acids, is used to synthesize acute phase protein and produce local energy by oxidation, or goes unused due to anabolic resistance [3, 17]. Plasma leucine concentrations may represent the net balance of these interacting mechanisms.

In the adaptive phase, substantial amounts of amino acids are needed for tissue repair and protein synthesis. Low leucine concentrations may indicate deficient amino acids and may be associated with impaired wound healing, tissue repair, muscle synthesis, and immune function [18]. Previously, in patients with COPD, Labaki et al. found that lower concentrations of serum leucine were independently associated with one-year incidence of respiratory exacerbation [19]. Labaki's observations are further supported by our data in the secondary analysis that, in patients who survived longer than two weeks after admitted in the ICU, lower leucine levels predicted a poorer weaning profile, longer length of ICU and hospital stays, and an even higher mortality rate compared to those with relatively normal plasma leucine concentrations. In our study, leucine < 109 *μ*M appeared to be associated with chronic malnutrition, since the values of all nutritional parameters were the lowest of the three subgroups. This notion was further supported by our finding that the prognostic value of leucine < 109 *μ*M disappeared after adjusting for albumin or other nutritional biomarkers. Whether aggressive supplementation of protein can improve the outcomes of these patients is an urgent matter in need of clarification.

In critical illness, a subgroup of patients develops anabolic resistance [20, 21], which is probably associated with insulin resistance, interstitial edema, and dysfunction of amino acid transporters on the cell membrane. In anabolic resistance, increased feeding of calories and protein does not give rise to increased protein synthesis. High plasma leucine levels may represent this metabolic phenotype, consistent with our previous report that high leucine levels in critically ill patients correlated with poor mitochondrial function leading to impaired beta-oxidation and increased unmetabolized acylcarnitines in the circulation [12]. High leucine levels may thus indicate a state of metabolic storm in response to critical stress. These conclusions are further supported by our current findings that leucine concentration > 174 *μ*M was associated with a higher incidence of mortality which mostly occurred during the acute phase within 2 weeks after being hospitalized to the ICU. Patients with leucine >174 *μ*M could not be easily distinguished from other patients by clinical characteristics, such as APACHE II and SOFA scores or laboratory parameters. Interestingly, although the leucine concentration was high in the circulation, the prealbumin level in these patients was low, which could mislead clinicians to attempt protein supplementation. Anabolic resistance might be mechanistically related to impaired prealbumin production. Although inadequate protein supplementation is associated with a poor prognosis [22], overfeeding is also harmful [23–26], and leucine concentration could help clinicians avoid making mistakes. These findings strongly imply that measuring leucine concentration in critically ill patients could differentiate the causes of malnutrition identified by traditional biomarkers.

### 4.1. Limitations

There are a few limitations in this study. First, although we and others have previously demonstrated significant prognostic value of leucine compared to other essential amino acids in critically ill patients [3, 12, 15, 18], it is possible that using a multiamino acid panel can provide additional value. However, doing so would negatively impact cost-benefit considerations. Second, for estimation of the patients survived longer than 2 weeks, since this was a secondary analysis, other nutritional biomarkers were not measured in the current study, such as albumin, prealbumin, and transferrin. To further confirm the independent prognostic value of low leucine level in this population, a prospective design should be designed in the future. Third, whether serial measurement of patients' leucine levels throughout the critical and subacute phases is helpful has not been proven and warrants further interventional investigation. Finally, we did not explore the prognostic value by combining leucine and other independent predictors of adverse outcomes in ICU. There is a strong need for improving outcome prediction by contemporary techniques such as machine learning models [27].

## 5. Conclusions

Our study demonstrated a U-shaped relationship between plasma leucine levels and mortality rate in patients admitted to the ICU due to critical status. Our findings provide a way to scientifically delineate the complex phases of catabolism and malnutrition initially defined by Cuthbertson et al. in 1950. Monitoring leucine levels could provide a meaningful reference to guide optimal nutrition support for patients hospitalized in the ICU.

## Figures and Tables

**Table 1:** Demographic and laboratory data in the initiation cohort.

	All	Survival	Death	*p* value
	*n* = 348	*n* = 232	*n* = 116	
Age (years)	71.4 ± 13.3	70.2 ± 13.9	73.7 ± 11.7	0.021
Male (%)	219 (62.9)	138 (59.5)	81 (69.8)	0.061
APACHE II score	20.2 ± 4.72	19.0 ± 3.77	22.5 ± 5.54	<0.001
SOFA score	7.07 ± 3.30	6.09 ± 2.77	9.05 ± 3.40	<0.001
Body mass index (kg/m^2^)	24.4 ± 4.98	24.7 ± 4.98	23.8 ± 4.94	0.108
Co-morbidity				
Diabetes mellitus (%)	176 (50.6)	117 (50.4)	59 (50.9)	1.000
Hypertension (%)	234 (67.2)	152 (65.5)	82 (70.7)	0.396
Coronary artery disease (%)	157 (45.1)	113 (48.7)	44 (37.9)	0.067
COPD (%)	27 (7.80)	24 (10.3)	3 (2.6)	0.010
Chronic kidney disease (%)	82 (23.6)	56 (24.1)	26 (22.4)	0.789
Ventilator use (%)	239 (68.7)	156 (67.2)	83 (71.6)	0.463
Inotropic agent use (%)	120 (34.5)	59 (25.4)	61 (52.6)	<0.001
Days in ICU (day)	12.2 ± 9.48	11.2 ± 8.82	14.0 ± 10.4	0.012
Days in hospital (day)	28.5 ± 22.8	29.3 ± 22.4	26.8 ± 23.6	0.326
Laboratory data				
White blood cell (1000/uL)	13.6 ± 8.19	13.2 ± 8.30	14.5 ± 7.91	0.152
Hemoglobin (g/dL)	11.0 ± 3.76	11.3 ± 3.01	10.6 ± 4.90	0.131
C-reactive protein (mg/L)	46.6 (11.4-103)	32.6 (7.52-81.4)	66.0 (26.6-148)	<0.001
Cholesterol (mg/dL)	134 ± 56.7	144 ± 61.5	115 ± 39.4	<0.001
Triglyceride (mg/dL)	112 (83.5-153)	112 (83.5-153)	112 (83.5-151)	0.981
eGFR (ml/min/1.73 m^2^)	33.5 (11.9-64.9)	38.6 (13.5-68.7)	24.0 (10.8-52.3)	0.086
ALT (U/L)	29 (17-60.7)	28.0 (16.0-54.0)	36.5 (18.0-100)	0.102
Albumin (g/dL)	3.24 ± 0.58	3.37 ± 0.55	2.99 ± 0.56	<0.001
Prealbumin (mg/dL)	15.3 ± 7.05	16.6 ± 7.06	12.5 ± 6.20	<0.001
Transferrin (mg/dL)	155 ± 49.0	164 ± 49.8	137 ± 42.5	<0.001
Leucine 109-174 *μ*M	143 (41.1)	107 (46.1)	36 (31.0)	0.008

Data are expressed as the mean ± SD for variables with normal distribution, median (interquartile range (IQR)) for variables with skewed distribution, and as number (percentage) for categorical variables. APACHE: acute physiology and chronic health evaluation; ALT: alanine aminotransferase; COPD: chronic obstructive pulmonary disease; chronic kidney disease: estimated glomerular filtration rate (eGFR) < 60 ml/min/1.73 m^2^; CRP: C-reactive protein; ICU: intensive care unit; SOFA: sequential organ failure assessment; WBC: white blood cell count.

**Table 2:** Comparisons of demographic and laboratory data in patients with different leucine concentrations in the initiation cohort.

	Leucine			
	109-174 *μ*M	<109 *μ*M	>174 *μ*M	*p* value^#^
	*n* = 143	*n* = 150	*n* = 55	
Age (years)	69.1 ± 13.9	74.0 ± 12.9	70.0 ± 11.5	0.552
Male (%)	96 (67.1)	81 (54)	42 (76.3)	0.015
APACHE II score	19.6 ± 4.17	20.7 ± 4.71^∗^	20.4 ± 5.89	0.041
SOFA score	6.55 ± 3.24	7.55 ± 3.25^∗∗^	7.11 ± 3.46	0.034
Body mass index (kg/m^2^)	24.8 ± 5.22	23.6 ± 5.54	24.8 ± 4.09	0.197
Admission to ICU	29 (20.2)	27 (18)	6 (10.9)	0.909
Comorbidity				
Diabetes mellitus (%)	69 (48.2)	71 (47.3)	36 (65.4)	0.165
Hypertension (%)	98 (82.1)	100 (66.6)	36 (65.4)	0.900
Coronary artery disease (%)	69 (48.2)	59 (39.3)	29 (52.7)	0.432
Atrial fibrillation (%)	21 (14.6)	28 (18.6)	10 (18.1)	0.640
COPD (%)	14 (9.79)	12 (8.0)	1 (1.8)	0.510
Chronic kidney disease (%)	37 (25.8)	31 (20.6)	14 (25.4)	0.540
Ventilator use (%)	96 (67.1)	109 (72.6)	34 (61.8)	0.873
Inotropic agent use (%)	37 (25.8)	64 (42.6)^∗∗^	19 (34.5)	0.012
Days in ICU (day)	10.8 ± 9.24	13.7 ± 9.24^∗^	11.5 ± 9.16	0.032
Days in hospital (day)	25.2 ± 520.9	32.3 ± 23.3^∗^	25.0 ± 24.6	0.007
Laboratory data				
White blood cell (1000/ul)	12.7 ± 5.47	14.1 ± 10.0	14.6 ± 8.32	0.221
Hemoglobin(g/dL)	11.7 ± 4.74	10.3 ± 2.69^∗^	11.4 ± 2.98	0.004
C-reactive protein (mg/L)	41.4 (8.73, 103)	45.2 (12.4, 105)	58.3 (14.6, 104)	0.760
Cholesterol (mg/dL)	142 ± 65.7	123 ± 46.1^∗^	142 ± 53.6^†^	0.010
Triglyceride (mg/dL)	119 (88, 156)	99 (77, 135)^∗^	126 (98, 180)^†^	0.003
eGFR (mL/min/1.73 m^2^)	44.5 ± 40.0	45.7 ± 43.1	43.2 ± 40.1	0.922
ALT (U/L)	27 (16, 49)	27 (16, 63.5)	43 (21, 134)^∗∗^^,†^	0.005
Albumin (g/dl)	3.40 ± 0.52	3.03 ± 0.58^∗^	3.38 ± 0.57^†^	<0.001
Prealbumin (mg/dL)	16.1 ± 6.66	14.4 ± 7.26	15.4 ± 7.23	0.087
Transferrin (mg/dL)	167 ± 50.4	146 ± 45.3^∗^	152 ± 46.3^†^	<0.001

Data are expressed as the *mean* ± *SD* for variables with normal distribution, as the median (interquartile range) for variables with skewed distribution, and as the number (percentage) for categorical variables. ^#^, comparisons by One-way ANOVA and Chi-square (multiple comparison with Bonferroni adjusted *p* value). ICU: intensive care unit; COPD: chronic obstructive pulmonary disease; eGFR: estimated glomerular filtration rate; CRP: C-reactive protein; ALT: alanine aminotransferase; SOFA: sequential organ failure assessment. ^∗^*p* < 0.05, ^∗∗^*p* < 0.01, compared to “Leucine 109-174 *μ*M”; ^†^*p* < 0.05, compared to “Leucine <109 *μ*M”.

**Table 3:** COX univariate and multivariable analysis of factors for predicting mortality in the initiation cohort.

	Univariate		Multivariable^∗^		Multivariable^†^	
	HR (95% CI)	*p* value	HR (95% CI)	*p* value	HR (95% CI)	*p* value
Leucine						
Leucine 109-174 *μ*M (reference)						
Leucine > 174 *μ*M	2.16 (1.30-3.59)	0.003	2.03 (1.22-3.37)	0.006	2.06 (1.22-3.47)	0.007
Leucine < 109 *μ*M	1.66 (1.10-2.51)	0.015	1.52 (1.01-2.31)	0.043	0.95 (0.60-1.50)	0.828
Age (years)	1.02 (1.00-1.03)	0.046				
APACHE II score	1.12 (1.09-1.16)	<0.001	1.08 (1.06-1.11)	<0.001	1.09 (1.05-1.12)	<0.001
SOFA score	1.23 (1.17-1.28)	<0.001	1.14 (1.07-1.21)	<0.001	1.18 (1.11-1.24)	<0.001
COPD	0.27 (0.09-0.86)	0.027				
C-reactive protein (log)	2.21 (1.62-3.01)	<0.001	2.26 (1.65-3.10)	<0.001		
Cholesterol (mg/dL)	0.99 (0.98-0.99)	<0.001	0.98 (0.98-0.99)	<0.001		
Albumin (g/dL)	0.392 (0.29-0.53)	<0.001	0.41 (0.29-0.54)	<0.001		
Prealbumin (mg/dL)	0.92 (0.89-0.95)	<0.001	0.92 (0.89-0.95)	<0.001		
Transferrin (mg/dL)	0.99 (0.98-0.99)	<0.001	0.98 (0.98-0.99)	<0.001		

APACHE: acute physiology and chronic health evaluation; CI: confidence interval; COPD: chronic obstructive pulmonary disease; HR: hazard ratio; SOFA: sequential organ failure assessment. ∗, adjusting for age and COPD. †, adjusting for age, COPD, C-reactive protein, cholesterol, albumin, prealbumin, and transferrin.

## Data Availability

The authors declare that the data underlying this article will be shared on reasonable request to the corresponding author.

## Supplementary Materials

Supplementary Materials Supplementary Table S1: demographic and laboratory data in the validation cohort. Supplementary Table S2: comparisons of demographic and laboratory data in patients with different leucine concentrations in the validation cohort. Supplementary Figure S1: an inverse-sigmoidal receiver operating characteristic curve for the leucine level to predict mortality. Supplemental Figure S2: the Kaplan-Meier curves for patients with leucine < 109 *μ*M, 109-174 *μ*M, >174 *μ*M (for all-cause death). Leucine levels were measured at baseline.

## References

[1] Vincent J.-L., Marshall J. C., Ñamendys-Silva S. A. (2014). Assessment of the worldwide burden of critical illness: the intensive care over nations (ICON) audit. *The Lancet Respiratory Medicine*.

[2] Knaus W. A., Draper E. A., Wagner D. P., Zimmerman J. E. (1985). APACHE II. *Critical Care Medicine*.

[3] Chen W.-S., Wang C.-H., Cheng C.-W. (2020). Elevated plasma phenylalanine predicts mortality in critical patients with heart failure. *ESC Heart failure*.

[4] Cuthebertson D. P., Tilstone W. J. (1950). Diet and trauma. *British Journal of Nutrition*.

[5] Wolfe R. R. (2018). The 2017 Sir David P Cuthbertson lecture. Amino acids and muscle protein metabolism in critical care. *Clinical Nutrition*.

[6] Lee S. H., Kim S. J., Choi Y. H., Lee J. H., Chang J. H., Ryu Y. J. (2018). Clinical outcomes and prognostic factors in patients directly transferred to the intensive care unit from long-term care beds in institutions and hospitals: a retrospective clinical study. *BMC Geriatrics*.

[7] Park J. E., Chung K. S., Song J. H. (2018). The C-reactive protein/albumin ratio as a predictor of mortality in critically ill patients. *Journal of Clinical Medicine*.

[8] Arabi Y. M., Aldawood A. S., al-Dorzi H. M. (2017). Permissive underfeeding or standard enteral feeding in high- and low-nutritional-risk critically ill Adults.Post HocAnalysis of the PermiT trial. *American Journal of Respiratory and Critical Care Medicine*.

[9] on behalf of the FROG-ICU study group, Lasocki S., Lefebvre T. (2018). Iron deficiency diagnosed using hepcidin on critical care discharge is an independent risk factor for death and poor quality of life at one year: an observational prospective study on 1161 patients. *Critical Care*.

[10] Meloun B., Morávek L., Kostka V. (1975). Complete amino acid sequence of human serum albumin. *FEBS Letters*.

[11] Kanda Y., Goodman D. S., Canfield R. E., Morgan F. J. (1974). The amino acid sequence of human plasma prealbumin. *The Journal of Biological Chemistry*.

[12] Wang C.-H., Cheng M.-L., Liu M.-H. (2018). Simplified plasma essential amino acid-based profiling provides metabolic information and prognostic value additive to traditional risk factors in heart failure. *Amino Acids*.

[13] Welsh P., Rankin N., Li Q. (2018). Circulating amino acids and the risk of macrovascular, microvascular and mortality outcomes in individuals with type 2 diabetes: results from the ADVANCE trial. *Diabetologia*.

[14] Hiraiwa H., Okumura T., Kondo T. (2021). Prognostic value of leucine/phenylalanine ratio as an amino acid profile of heart failure. *Heart and Vessels*.

[15] Huang S.-S., Lin J.-Y., Chen W.-S. (2019). Phenylalanine- and leucine-defined metabolic types identify high mortality risk in patients with severe infection. *International Journal of Infectious Diseases*.

[16] Vincent J. L., Moreno R., Takala J. (1996). The SOFA (sepsis-related organ failure assessment) score to describe organ dysfunction/failure. *Intensive Care Medicine*.

[17] Moyer E., Cerra F., Peters D. (1981). Multiple systems organ failure. *The Journal of Trauma*.

[18] Nie C., He T., Zhang W., Zhang G., Ma X. (2018). Branched chain amino acids: beyond nutrition metabolism. *International Journal of Molecular Sciences*.

[19] Labaki W. W., Gu T., Murray S. (2019). Serum amino acid concentrations and clinical outcomes in smokers: SPIROMICS metabolomics study. *Scientific Reports*.

[20] Biolo G., Fleming R. Y., Maggi S. P., Nguyen T. T., Herndon D. N., Wolfe R. R. (2002). Inverse regulation of protein turnover and amino acid transport in skeletal muscle of hypercatabolic patients. *The Journal of Clinical Endocrinology and Metabolism*.

[21] Miller S., Chinkes D., MacLean D. A., Gore D., Wolfe R. R. (2004). In vivo muscle amino acid transport involves two distinct processes. *American Journal of Physiology Endocrinology and Metabolism*.

[22] Weijs P. J., Sauerwein H. P., Kondrup J. (2012). Protein recommendations in the ICU: g protein/kg body weight - which body weight for underweight and obese patients?. *Clinical Nutrition*.

[23] Reid C. (2006). Frequency of under- and overfeeding in mechanically ventilated ICU patients: causes and possible consequences. *Journal of Human Nutrition and Dietetics*.

[24] Krishnan J. A., Parce P. B., Martinez A., Diette G. B., Brower R. G. (2003). Caloric intake in medical ICU patients: consistency of care with guidelines and relationship to clinical outcomes. *Chest*.

[25] McClave S. A., Taylor B. E., Martindale R. G. (2016). Guidelines for the provision and assessment of nutrition support therapy in the adult critically ill Patient. *Journal of Parenteral and Enteral Nutrition*.

[26] Levy M. M., Artigas A., Phillips G. S. (2012). Outcomes of the surviving sepsis campaign in intensive care units in the USA and Europe: a prospective cohort study. *The Lancet Infectious Disease*.

[27] Barchitta M., Maugeri A., Favara G. (2021). Early prediction of seven-day mortality in intensive care unit using a machine learning model: results from the SPIN-UTI Project. *Journal of Clinical Medicine*.

