# Supplementary material for: U-Shape Relationship between Plasma Leucine Level and Mortality in the Intensive Care Unit
Source: Dis Markers. 2022 Jan 7;2022:7389258. doi: 10.1155/2022/7389258 (PMC8759908; doi:10.1155/2022/7389258)

**Supplemental material**

# U-shape relationship between plasma leucine level and mortality in the intensive care unit

Mei-Ying Wang, MD^1,3^Chao-Hung Wang, MD, PhD ^1,2^; Wei-Siang Chen, MD^1,3^; Chien-Ming Chu, MD^4^; Huang-Ping Wu, MD^4^; Min-Hui Liu, RN, NP, PhD ^1,5^; Yi-Tsen Lin, MSc ^6^; Kuo-Chin Kao, MD ^2,7^; Chung-Yu Liang, MD^1,3^; Wen-Hsin Chen, MD^1,3^; Huei-Jen Wang, RN, BSN ^5^; Shu-Chiu Lee, RN, MSN^5^

^1^ Heart Failure Research Center, Division of Cardiology, Department of Internal Medicine, Chang Gung Memorial Hospital, 20401 Keelung, Taiwan

^2^ Chang Gung University College of Medicine, 33302 Taoyuan, Taiwan

^3^ Intensive Care Unit, Division of Cardiology, Department of Internal Medicine, Chang Gung Memorial Hospital, 20401 Keelung, Taiwan

^4^ Division of pulmonary, critical care and sleep medicine, Chang Gung Memorial Hospital, 20401 Keelung, Taiwan

^5^ Department of Nursing, Keelung Chang Gung Memorial Hospital, 20401 Keelung, Taiwan

^6^ Nutrition department, Chang Gung Memorial Hospital, 20401 Keelung, Taiwan

^7^ Department of Thoracic Medicine, Chang Gung Memorial Hospital, 33305 Taoyuan, Taiwan

**Correspondence should be addressed to:**

Chao-Hung Wang, MD, PhD,

Heart Failure Research Center,

Division of Cardiology, Department of Internal Medicine,

Chang Gung Memorial Hospital,

222 Mai Chin Road, Keelung, Taiwan

Tel. 886 2 24313131, ext. 2245

E-mail: [bearty54@gmail.com](mailto:bearty54@gmail.com); bearty@cgmh.org.tw

| **Supplemental Table S1.** Demographic and laboratory data in the validation cohort | | | | | | |
| --- | --- | --- | --- | --- | --- | --- |
|  |  | All |  | Survival | Death |  |
|  |  | n = 106 |  | n =69 | n = 37 | *p* value |
| Age (years) |  | 72.2±13.8 |  | 71.6±14.7 | 73.4±12.0 | 0.510 |
| Male (%) |  | 65(61.3) |  | 41(59.4) | 24(64.9) | 0.583 |
| APACHE II score |  | 19.1±4.88 |  | 17.8±3.3.78 | 21.4±5.83 | 0.001 |
| SOFA score |  | 7.14±3.67 |  | 6.07±3.28 | 9.14±3.56 | <0.001 |
| Body mass index (kg/m^2^) |  | 25.0±4.82 |  | 24.0±3.75 | 26.7±6.05 | 0.006 |
| Co-morbidity |  |  |  |  |  |  |
| Diabetes mellitus (%) |  | 48(45.3) |  | 32(46.4) | 16(43.2) | 0.757 |
| Hypertension (%) |  | 63(59.4) |  | 39(56.5) | 24(64.9) | 0.404 |
| Coronary artery disease (%) |  | 26(24.5) |  | 15(21.7) | 11(29.7) | 0.362 |
| COPD (%) |  | 4(3.8) |  | 4(5.8) | 0(0) | 0.135 |
| Chronic kidney disease (%) |  | 36(34.0) |  | 23(33.3) | 213(35.1) | 0.852 |
| Ventilator use (%) |  | 77(72.6) |  | 45(65.2) | 32(86.5) | 0.019 |
| Inotropic agent use (%) |  | 46(43.4) |  | 25(36.2) | 21(56.8) | 0.042 |
| Days in ICU (day) |  | 11.3±10.3 |  | 10.0±7.34 | 13.6±14.2 | 0.086 |
| Days in hospital (day) |  | 26.5±22.9 |  | 26.7±21.8 | 26.1±25.1 | 0.898 |
| Laboratory data |  |  |  |  |  |  |
| White blood cell (1000/uL) |  | 13.1±6.68 |  | 12.4±6.61 | 14.4±6.69 | 0.138 |
| Hemoglobin (g/dL) |  | 10.4±2.72 |  | 10.4±2.72 | 10.4±2.77 | 0.989 |
| C-reactive protein (mg/L) |  | 41.5(13.2-128) |  | 33.1(9.21-107) | 49.6(25.3-144) | 0.090 |
| Cholesterol (mg/dL) |  | 130±47.2 |  | 140±46.0 | 110±43.4 | 0.001 |
| Triglyceride (mg/dL) |  | 108(78.5-152) |  | 106(81.5-164) | 112(82-146) | 0.615 |
| eGFR (ml/min/1.73 m^2^) |  | 36.9(14.9-68.1) |  | 38.2(19.4-73.5) | 34.7(9.12-56.1) | 0.220 |
| ALT (U/L) |  | 33.5(17-68.7) |  | 32(16.5-70.5) | 31(20-63) | 0.884 |
| Albumin (g/dL) |  | 3.08±0.63 |  | 3.23±0.59 | 2.80±0.61 | 0.001 |
| Pre-Albumin (mg/dL) |  | 14.1±6.83 |  | 15.5±7.17 | 11.6±5.33 | 0.004 |
| Transferrin (mg/dL) |  | 149±49.6 |  | 163±45.6 | 124±46.8 | <0.001 |
| Leucine 109-174 μM |  | 65(61.3) |  | 37(53.6) | 28(75.7) | 0.026 |

Data are expressed as the mean ± SD for variables with normal distribution, median [interquartile range (IQR)] for variables with skewed distribution, and as number (percentage) for categorical variables. APACHE, acute physiology and chronic health evaluation; ALT, alanine aminotransferase; COPD, chronic obstructive pulmonary disease; chronic kidney disease, estimated glomerular filtration rate (eGFR) < 60 ml/min/1.73 m^2^; CRP, C-reactive protein; ICU, intensive care unit; SOFA, sequential organ failure assessment; WBC, white blood cell count.

**Supplemental Table S2.** Comparisons of demographic and laboratory data in patients with different leucine concentrations in the validation cohort

|  |  | Leucine |  |  |
| --- | --- | --- | --- | --- |
|  | 109-174 μM | <109 μM | >174 μM |  |
|  | n = 41 | n = 47 | n = 18 | *p* value ^#^ |
| Age (years) | 73.3±14.4 | 73.0±13.6 | 68.1±12.9 | 0.365 |
| Male (%) | 30(73.2) | 24(51.1) | 11(61.1) | 0.105 |
| APACHE II score | 16.5±6.04 | 18.5±5.65 | 18.9±7.25 | 0.230 |
| SOFA score | 6.78±3.53 | 7.06±3.75 | 8.17±3.81 | 0.406 |
| Body mass index (kg/m^2^) | 25.4±4.58 | 23.8±4.62 | 27.3±5.19^††^ | 0.024 |
| Co-morbidity |  |  |  |  |
| Diabetes mellitus (%) | 18(43.9) | 22(46.8) | 8(44.4) | 0.960 |
| Hypertension (%) | 26(63.4) | 28(59.6) | 9(50.0) | 0.627 |
| Coronary artery disease (%) | 19(46.3) | 10(21.3)^*^ | 9(50.0) ^†^ | 0.020 |
| Atrial fibrillation (%) | 4(9.8) | 42(10.6) | 17(5.6) | 0.818 |
| COPD (%) | 3(7.3) | 0(0.0) | 1(5.6) | 0.181 |
| Chronic kidney disease (%) | 16(39.0) | 17(36.2) | 3(16.7) | 0.226 |
| Ventilator use (%) | 26(63.4) | 30(63.8) | 16(88.9) | 0.112 |
| Inotropic agent use (%) | 16(39.0) | 20(42.6) | 10(55.6) | 0.493 |
| Days in ICU (day) | 10.9±10.8 | 11.5±10.3 | 11.7±10.1 | 0.942 |
| Days in hospital (day) | 22.1±15.5 | 32.7±28.8 | 21.1±16.7 | 0.051 |
| Laboratory data |  |  |  |  |
| White blood cell (1000/ul) | 11.9±6.38 | 12.7±6.10 | 16.9±7.79^**,†^ | 0.026 |
| Hemoglobin(g/dL) | 10.9±2.71 | 10.1±2.53 | 10.3±3.24 | 0.374 |
| C-reactive protein (mg/L) | 27.7(13.6-113) | 53.2(16.1-129) | 24.5(2.38-107) | 0.354 |
| Cholesterol (mg/dL) | 133±45.8 | 123±37.6 | 142±68.4 | 0.305 |
| Triglyceride (mg/dL) | 138±99.5 | 105±40.6 | 186±115^*,††^ | 0.002 |
| eGFR(mL/min/1.73m^2^) | 42.0±36.8 | 50.8±41.7 | 34.3±23.8 | 0.243 |
| ALT (U/L) | 35.0(17.5-80.5) | 35.0(19.0-55.0) | 29.0(11.8-134) | 0.784 |
| Albumin (g/dl) | 3.27±0.56 | 3.01±0.58 | 2.86±0.83^*^ | 0.038 |
| Pre-Albumin (mg/dL) | 16.1±7.03 | 12.7±6.91 | 13.7±5.27 | 0.064 |
| Transferrin (mg/dL) | 160±39.0 | 140±51.3 | 150±63.5 | 0.166 |

Data are expressed as the mean ± SD for variables with normal distribution, as the median [interquartile range] for variables with skewed distribution, and as the number (percentage) for categorical variables. ^#^, comparisons by One-way ANOVA and Chi-square (multiple comparison with Bonferroni adjusted p value). ICU, intensive care unit; COPD, chronic obstructive pulmonary disease; eGFR, estimated glomerular filtration rate; CRP, C-reactive protein; ALT, alanine aminotransferase; SOFA, sequential organ failure assessment. ^*^*p*<0.05, ^**^*p*<0.01, compared to “Leucine 109-174 μM”; ^†^*p*<0.05, ^††^*p*<0.01, compared to “Leucine <109 μM”.

**Supplemental Figures:**

**Supplemental Figure S1.** An inverse-sigmoidal receiver operating characteristic curve for the leucine level to predict mortality. Arrows indicate the cutoff values.


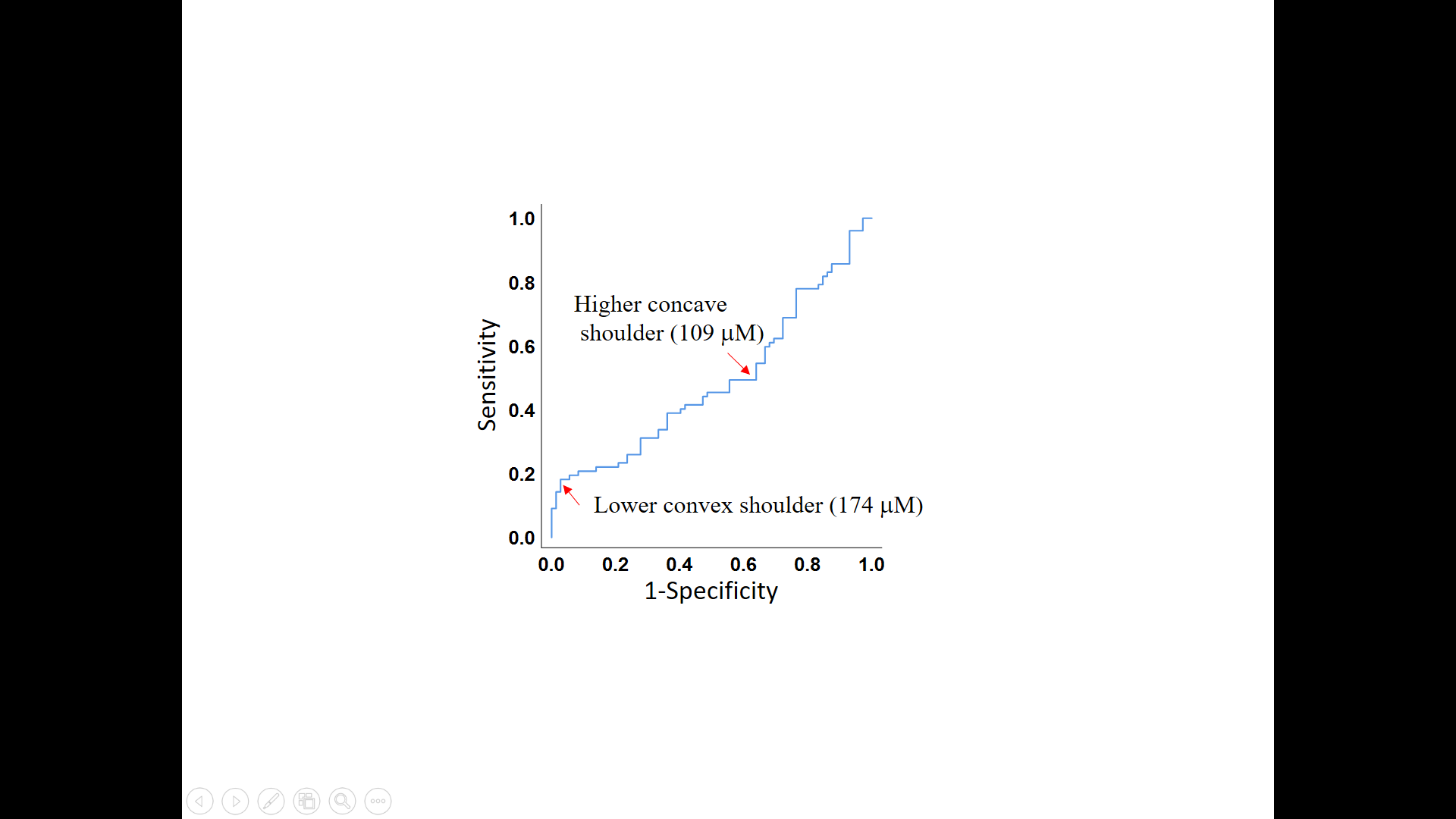


**Supplemental Figure S2.** The Kaplan-Meier curves for patients with leucine <109 μM, 109-174 μM, >174 μM (for all-cause death). Leucine levels were measured at baseline


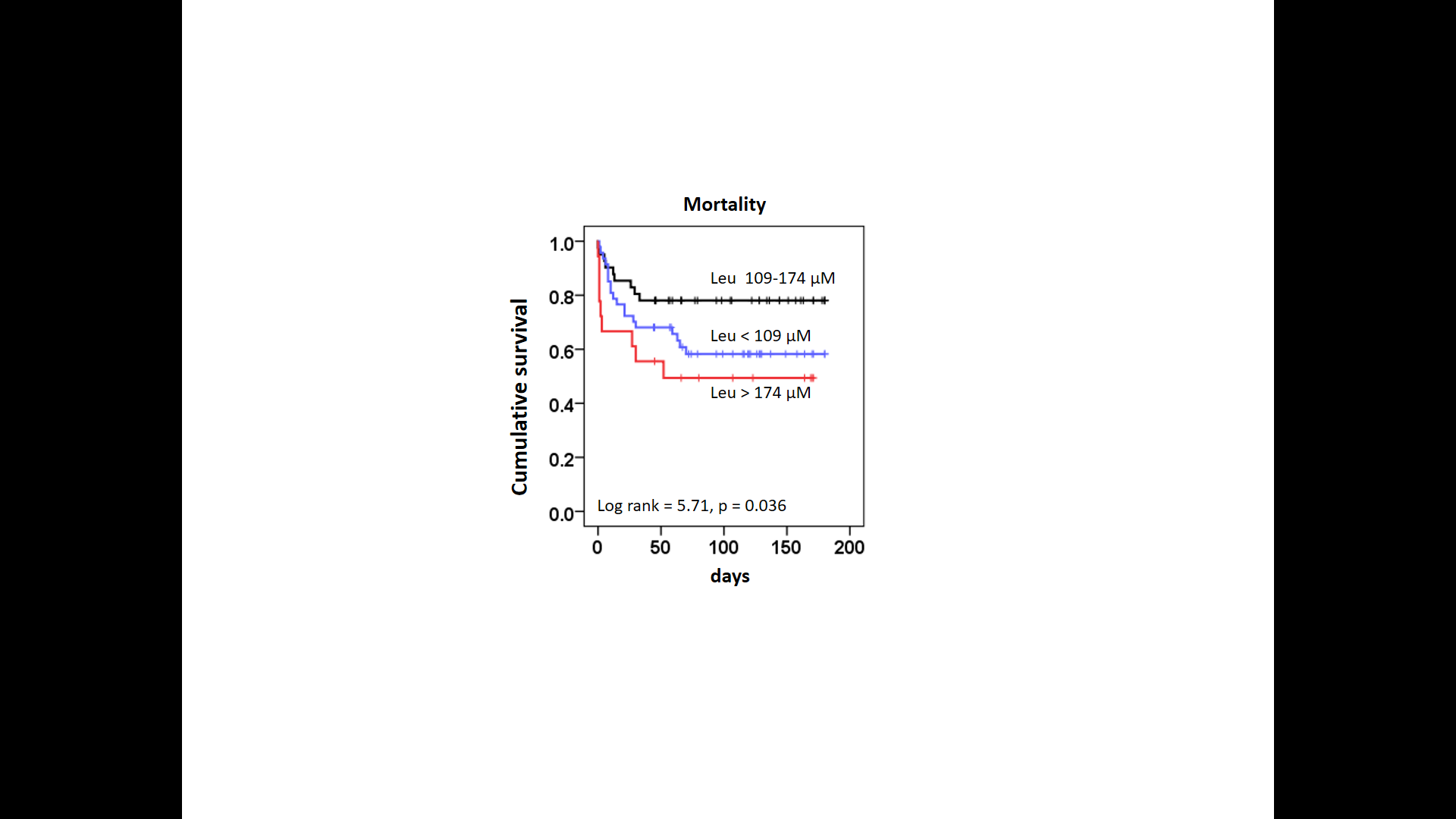

Supplement: Supplementary Materials — Supplementary Table S1: demographic and laboratory data in the validation cohort. Supplementary Table S2: comparisons of demographic and laboratory data in patients with different leucine concentrations in the validation cohort. Supplementary Figure S1: an inverse-sigmoidal receiver operating characteristic curve for the leucine level to predict mortality. Supplemental Figure S2: the Kaplan-Meier curves for patients with leucine < 109 μM, 109-174 μM, >174 μM (for all-cause death). Leucine levels were measured at baseline. [file 7389258.f1.doc]
